# Effects of Austenitizing Temperature and Deep Cryogenic Treatment on Microstructural Evolution and Mechanical Properties of a Microalloyed High-Carbon Steel

**DOI:** 10.3390/ma19071342

**Published:** 2026-03-28

**Authors:** Jian Zhang, Chenglian Zhang, Han Dong

**Affiliations:** 1 School of Materials Science and Engineering, Shanghai University, Shanghai 200444, China; 2 State Key Laboratory of Advanced Special Steel, Shanghai University, Shanghai 200444, China; 3 Shandong Zhongke Xiwang New Material Technology Research and Development Co., Ltd., Zouping 256209, China; zcl311@163.com

**Keywords:** microalloyed high-carbon steel, DCT, microstructural evolution, mechanical property, austenitizing temperature

## Abstract

A microalloyed high-carbon low-alloy steel was designed to clarify the combined effects of austenitizing temperature and deep cryogenic treatment (DCT) on microstructural evolution and mechanical performance. Specimens were austenitized at 770–900 °C, water-quenched, subjected to DCT at −196 °C, and subsequently tempered at 180 °C. Microstructural characterization by XRD, EBSD, and TEM indicates that the quenched microstructure is dominated by martensite and cementite, with retained austenite below 1% at moderate austenitizing temperatures. DCT does not fundamentally alter the martensitic morphology but promotes the transformation of retained austenite and induces substructure fragmentation, dislocation reorganization, and a more homogeneous lattice strain distribution. Concurrently, carbon redistribution during cryogenic exposure facilitates the formation of finely dispersed carbides. After tempering, partial recovery and stabilization of the martensitic substructure lead to reduced lattice distortion while maintaining a high density of effective strengthening features. Mechanical testing shows that DCT combined with appropriate austenitizing (770–790 °C) improves hardness and ultimate tensile strength with acceptable ductility, whereas excessive austenitizing at 900 °C results in severe grain coarsening and intergranular brittle fracture. The results demonstrate that optimized integration of microalloying and DCT enables a favorable strength–toughness balance in high-carbon tool steels.

## 1. Introduction

High-carbon low-alloy tool steels are widely used in files, saw blades, taps, and gauges due to their high hardness, excellent wear resistance, and relatively low alloying cost [1,2,3]. Under equilibrium conditions, these steels contain a high volume fraction of finely dispersed cementite (Fe_3_C), which underpins their service performance. However, their low alloying level also leads to several inherent limitations. The absence of strong carbide-forming elements shifts the continuous cooling transformation (CCT) curves to shorter times and increases the critical cooling rate, often necessitating water quenching to obtain full martensite [4,5]. Such severe cooling induces high thermal and transformation stresses, increasing distortion and quench cracking risks [6]. Moreover, poor hot hardness above ~200 °C results in rapid tempering softening of martensite, degrading wear resistance [7,8]. The presence of retained austenite further compromises hardness and dimensional stability, as its decomposition during service or tempering may cause dimensional variation and performance deterioration [9,10,11].

Microalloying has emerged as an effective strategy to overcome these limitations [10,12,13]. The addition of trace elements such as Nb [14,15], V [16,17], Ti [18], Mo [17], or Cr [10] enables control of austenite grain growth, phase transformation, and precipitation behavior without significantly increasing cost [19]. Fine carbides or carbonitrides formed by these elements inhibit grain coarsening, stabilize cementite morphology, and suppress coarse or networked Fe_3_C formation [20], thereby improving microstructural uniformity and reducing the critical cooling rate [21].

In addition, with respect to heat treatment optimization, deep cryogenic treatment (DCT) has emerged as an important supplement to the conventional “quenching & tempering” route, and has attracted increasing attention in recent years [22,23,24,25]. DCT is typically conducted at liquid nitrogen temperature (−196 °C), and its strengthening mechanism extends far beyond the simple promotion of retained austenite transformation to martensite [26,27,28]. Previous studies have demonstrated that the severe thermal contraction and martensitic transformation during cryogenic treatment introduce high strain energy into the microstructure, providing a strong driving force for carbon atom migration, segregation, and redistribution within the supersaturated martensitic matrix [29,30]. This process induces the precipitation of a high density of nanoscale transition carbides, such as ε-carbide [29,31,32]. These finely dispersed nano-precipitates effectively pin dislocations and generate pronounced precipitation strengthening, playing a critical role in enhancing hardness and wear resistance [33]. Moreover, DCT can partially relieve localized residual stresses and promote a more thermodynamically stable microstructure, thereby exerting a limited but beneficial influence on toughness while maintaining high strength [34,35,36].

However, the high internal stresses introduced by DCT may also increase the risk of microstructural embrittlement if not properly controlled. Consequently, low-temperature tempering after DCT (typically in the range of 150–200 °C) is considered an indispensable stabilization step. This process not only relieves part of the cryogenically induced internal stress but also promotes the stabilization of transition carbides and transforms as-quenched brittle martensite into tempered martensite with improved toughness. Through this route, a more favorable balance among hardness, wear resistance, toughness, and dimensional stability can be achieved [37,38].

It is noteworthy that although both microalloying and DCT have been demonstrated to effectively tailor microstructure and enhance performance in various steel grades, systematic investigations focusing on the combined effects of austenitizing parameters and DCT in microalloyed high-carbon steels remain scarce. In particular, the influence of austenitizing temperature on retained austenite stability, cryogenic transformation behavior, and subsequent tempering-induced precipitation processes has not yet been fully clarified. Therefore, a microalloyed high-carbon steel was selected as the research object to systematically investigate the effects of austenitizing temperature and DCT on microstructural evolution and mechanical properties. This work aims to provide new practical guidance for overcoming the performance limitations of high-carbon tool steels and improving their service reliability.

## 2. Materials and Methods

### 2.1. Chemical Composition and Initial Microstructure of the Experimental Materials

The experimental steel was produced using vacuum induction melting (VIM) to ensure high purity and precise control over its chemical composition. The nominal chemical composition is shown in Table 1. The as-cast ingot was subsequently homogenized and then hot rolled after heating to 1100–1150 °C. Multi-pass hot rolling was carried out starting from 1100–1050 °C, reducing the diameter to 20 mm. The final rolling temperature was 850 °C, followed by rapid cooling to 700 °C to prevent the formation of network-like carbides. After cooling to 700 °C, the steel was buried in sand for slow cooling to avoid the formation of thermal stress cracks. The spheroidizing annealing process involved heating at 760–770 °C for 2–4 h, followed by isothermal holding at 680–700 °C for 4–6 h. The furnace was then cooled further to 500–600 °C before the final air cooling to obtain a uniform initial microstructure suitable for further heat treatment.

**Figure 1:**
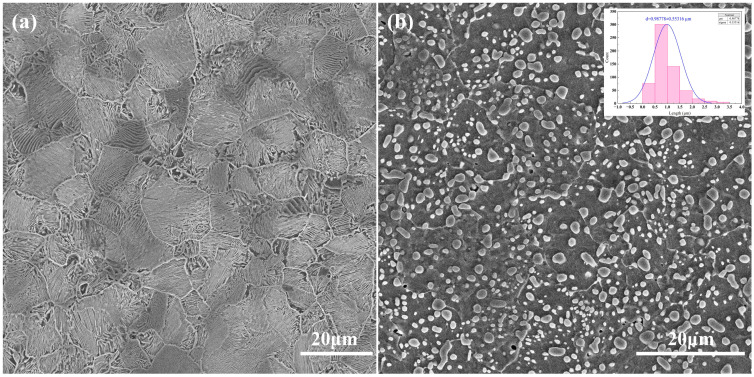
Microstructure of the experimental steel as-received: (**a**) Hot-rolled lamellar pearlite; (**b**) Spheroidal pearlite after spheroidizing annealing.

The initial microstructures of the experimental steel are presented in Figure 1. Figure 1a reveals that the as-hot-rolled microstructure consists of a typical lamellar pearlite, within which the prior austenite grain boundaries are discernible, yielding an average grain size of approximately 15 μm. The lamellar cementite is uniformly distributed between ferrite lamellae, indicating complete pearlite transformation during controlled cooling. After spheroidizing annealing, the lamellar cementite transforms into fine, uniformly distributed spheroidal carbides, as illustrated in Figure 1b. Statistical analysis reveals that the average carbide size after spheroidization is about 0.98 μm. The transformation from lamellar to spheroidal carbides effectively reduces the internal stress and improves the structural stability of the steel, thereby providing a more homogeneous and thermodynamically stable initial microstructure for subsequent heat treatment.

### 2.2. Critical Temperature Determination and Heat Treatment Process

**Figure 2:**
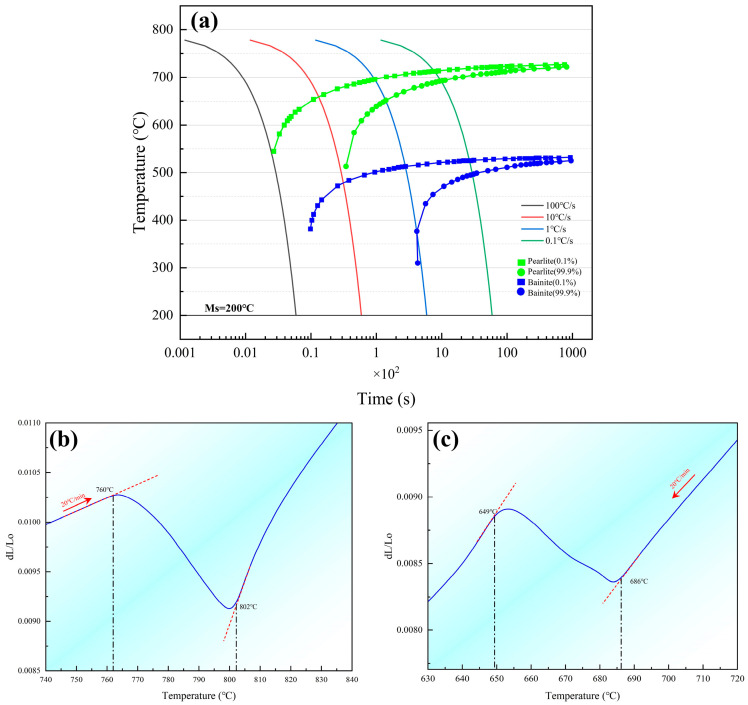
(**a**) Calculated CCT diagram; (**b**,**c**) dilatometric curves during heating and cooling of the test steel showing the critical transformation temperatures.

The continuous cooling transformation (CCT) diagram of the investigated steel was calculated using JMatPro software (Version 7.0), as shown in Figure 2a. The critical cooling rate to suppress pearlite formation was determined to be 100 °C/s, and the martensite start (Ms) temperature was predicted to be 200 °C. The austenitization critical temperatures of the experimental steel were determined using a DIL 402 Expedis Select dilatometer (Netzsch, Selb, Germany). The specimen was heated to 1000 °C at a rate of 20 °C/min, held for 5 min, and then cooled to room temperature at the same rate, while continuously recording both temperature and dimensional changes. The dilatometric curve obtained during continuous heating and cooling is shown in Figure 2b,c. By applying the tangent method to this curve, the austenite transformation start temperature (Ac1) and the finish temperature (Accm, corresponding to the complete dissolution of cementite) were identified as 760 °C and 802 °C, respectively. (see Figure 2b). Furthermore, the cooling segment of the cycle revealed the critical temperatures upon cooling, with Arcm (the start temperature for carbide precipitation from austenite) and Ar1 (the austenite-to-pearlite transformation start temperature) determined to be 686 °C and 649 °C, respectively (as shown in Figure 2c).

**Figure 3:**
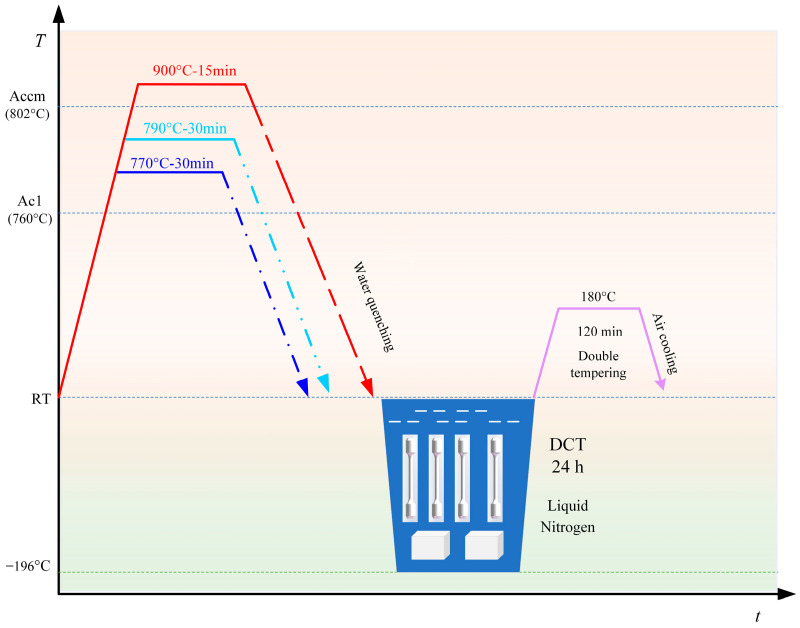
Schematic diagram of the heat treatment process.

To investigate the influence of heat treatments on the microstructure and mechanical properties of the steel, the heat treatment procedures, as illustrated in Figure 3, were designed. The specimens were austenitized at 770 °C, 790 °C, and 900 °C for a predetermined holding time and followed by water quenching. And then, the specimens were subjected to DCT by immersion in liquid nitrogen (−196 °C) for 24 h after quenching. Subsequently, the samples were removed and allowed to return gradually to room temperature before tempering at 180 °C. To facilitate a clearer and more intuitive identification of specimens subjected to different heat-treatment routes, the samples are designated as follows: specimens subjected to quenching only are denoted as Q; specimens treated by quenching followed by deep cryogenic treatment (DCT) are referred to as QC; specimens treated by quenching followed by tempering are designated as QT; and specimens subjected to quenching, subsequent DCT, and final tempering are denoted as QCT. Thereafter, these abbreviations are used consistently throughout the manuscript.

### 2.3. Microstructural Characterization and Mechanical Properties Test

For microstructural characterization, metallographic samples were prepared through a standard procedure involving sequential grinding with SiC abrasive papers followed by polishing with 1.5 μm diamond suspension to achieve a mirror finish. After ultrasonic cleaning, the polished specimens were chemically etched with 4% nitric acid alcohol etching solution to reveal the microstructure. Phase identification was performed using a Bruker D8 Advance X-ray diffractometer (XRD, Bruker AXS, Karlsruhe, Germany) with Cu Kα radiation (λ = 1.5406 Å), employing a step size of 0.02° and a scanning rate of 2°/min. Microstructural examination was carried out using a TESCAN VEGA3 SBH (Tescan, Brno, Czech) scanning electron microscope equipped with an Oxford Instruments Ultim Max energy dispersive spectroscopy (EDS, Oxford, UK) system. For crystallographic analysis, electron backscatter diffraction (EBSD) was performed using a NOVA NANOSEM 450 field-emission scanning electron microscope (SEM, FEI, OR, USA) equipped with an Oxford Symmetry detector. Transmission electron microscopy (TEM, FEI, OR, USA) investigation was conducted on a FEI Tecnai F20 field-emission operating at 200 kV. Thin foil specimens for TEM observation were prepared by mechanical grinding followed by ion milling, and were analyzed using bright-field imaging and selected-area electron diffraction (SAED).

**Figure 4:**
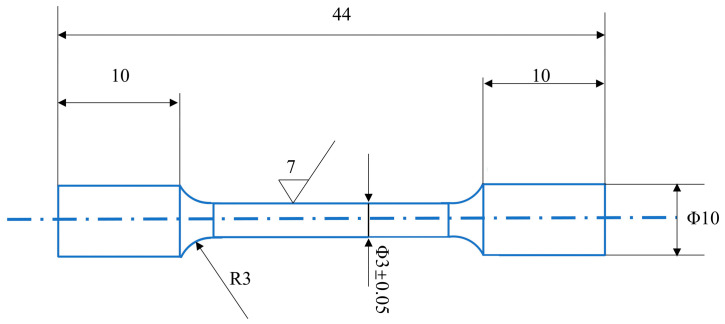
Schematic diagram of tensile specimen dimensions.

Mechanical properties were evaluated through tensile testing using a Shimadzu AGX-V (Shimadzu, Kyoto, Japan) universal testing machine with TRViewX digital image correlation system for strain measurement. The geometry of tensile specimens is illustrated in the accompanying Figure 4. Fractographic analysis of broken tensile specimens was performed using the TESCAN VEGA3 SBH scanning electron microscope (Tescan, Brno, Czech Republic). Hardness measurements were obtained using an HR-150 Rockwell hardness tester with a load of 150 kg applied for 15 s. The reported hardness values represent the average of at least five valid measurements after excluding outliers.

## 3. Results

### 3.1. Effects of Heat Treatments on Microstructural Evolution

**Figure 5:**
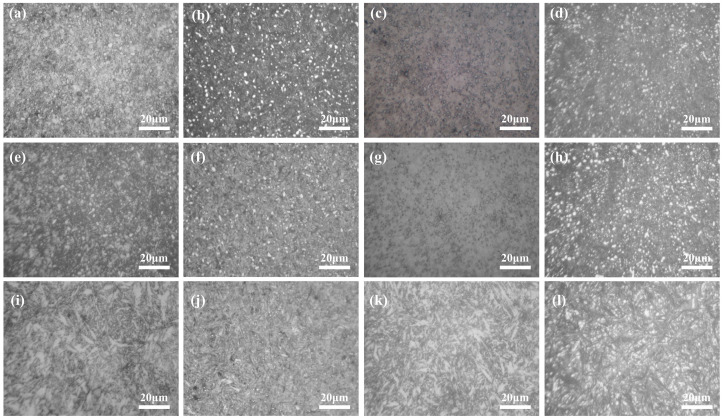
Optical microscope microstructure after different treatments: (**a**) 770 °C Q; (**b**) 770 °C QT; (**c**) 770 °C QC; (**d**) 770 °C QCT; (**e**) 790 °C Q; (**f**) 790 °C QT; (**g**) 790 °C QC; (**h**) 790 °C QCT; (**i**) 900 °C Q; (**j**) 900 °C QT; (**k**) 900 °C QC; (**l**) 900 °C QCT.

**Figure 6:**
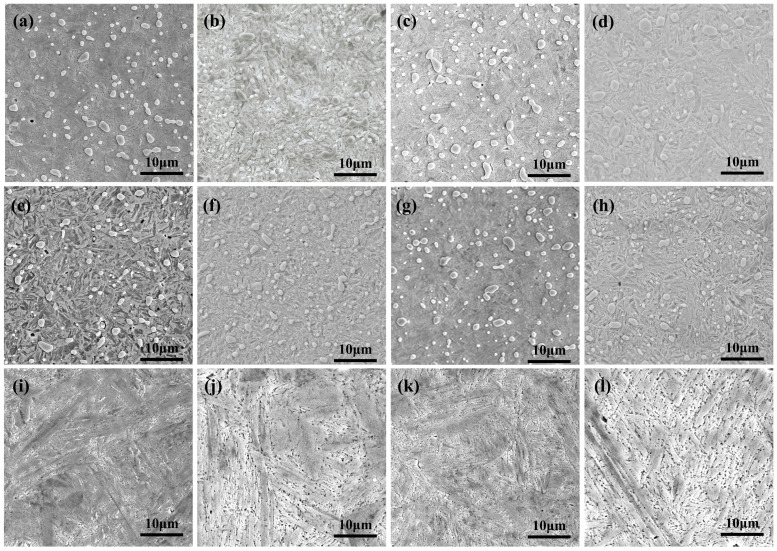
SEM microstructure after different treatments: (**a**) 770 °C Q; (**b**) 770 °C QT; (**c**) 770 °C QC; (**d**) 770 °C QCT; (**e**) 790 °C Q; (**f**) 790 °C QT; (**g**) 790 °C QC; (**h**) 790 °C QCT; (**i**) 900 °C Q; (**j**) 900 °C QT; (**k**) 900 °C QC; (**l**) 900 °C QCT.

Figure 5 and Figure 6 illustrate the microstructural evolution of the microalloyed high-carbon steel subjected to quenching and subsequent DCT at different austenitizing temperatures. At austenitizing temperatures of 770 °C and 790 °C, the quenched microstructure is mainly composed of fine lath martensite and undissolved cementite. With increasing austenitizing temperature, a slight coarsening of martensite laths is observed, which can be primarily attributed to the growth of prior austenite grains and the enhanced dissolution of carbides during austenitization [39]. Meanwhile, as the austenitizing temperature increases, the degree of carbide dissolution becomes more pronounced, resulting in a reduction in the average size of undissolved carbides. No significant alteration in the overall microstructural morphology is detected after DCT. However, a noticeable increase in the population of fine carbide particles is evident within the martensitic matrix. This observation aligns with established literature [32,40], which indicates that DCT not only promotes the transformation of retained austenite into martensite but also facilitates the precipitation of finely dispersed secondary carbides [41].

When the austenitizing temperature is increased to 900 °C, the quenched microstructure becomes significantly coarser, characterized by wider martensite laths and a higher fraction of retained austenite. Although DCT still promotes partial transformation of retained austenite, its refinement effect is limited under this condition. This suggests that excessive austenitizing temperature leads to overgrown prior austenite grains, which weakens the microstructural control capability of DCT.

**Figure 7:**
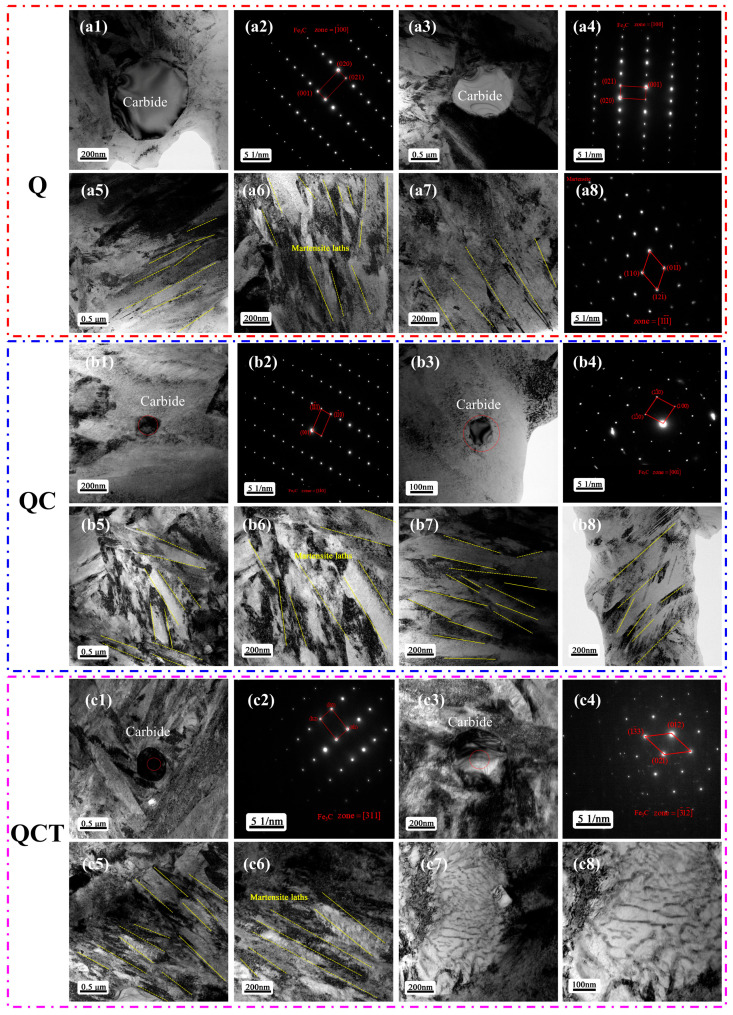
TEM images of the experimental steel subjected to different processing routes following austenitizing at 790 °C. (**a1**–**a8**) Microstructure after quenching. (**b1**–**b8**) Microstructure after quenching + DCT. (**c1**–**c8**) Microstructure after quenching + DCT + tempering.

To further elucidate the microstructural evolution of the experimental steel subjected to different heat-treatment routes, detailed TEM investigations were conducted. Figure 7 presents the TEM microstructures of the experimental steel quenched at 790 °C, followed by DCT and subsequent low-temperature tempering. As revealed by the TEM observations, the carbides present in the steel after different heat treatments, including Q, QC, and QCT, are predominantly identified as cementite (Fe_3_C), while no other types of alloy carbides or carbonitrides are detected. This indicates that, despite the introduction of microalloying elements, the strengthening contribution of secondary alloy carbides remains limited under the present heat-treatment conditions. Instead, the microstructural evolution and strengthening behavior are mainly governed by martensitic transformation, dislocation substructure development, and cementite-related precipitation processes.

As shown in Figure 7(a5–a7), in the quenched condition, the martensitic microstructure is characterized by relatively coarse lath martensite with straight, continuous, and well-defined lath boundaries, reflecting the high carbon content of the steel and the relatively elevated austenitizing temperature. The average martensite lath width in the quenched state is approximately 180 nm, indicating a comparatively low density of internal substructures and limited plastic accommodation within the martensitic matrix.

After DCT, the martensitic laths exhibit a pronounced tendency toward refinement and partial fragmentation, accompanied by a significant increase in dislocation density. The average martensite lath width is reduced to approximately 134 nm, demonstrating the strong refinement effect induced by cryogenic exposure. This feature is particularly evident in Figure 7(b5–b8), where dense dislocation tangles, dislocation walls, and localized lattice distortion are clearly observed. Such microstructural characteristics are typically associated with intense transformation-induced stresses, severe lattice contraction, and volumetric strain during DCT, which not only promote the transformation of retained austenite into martensite but also lead to substantial accumulation of plastic strain within the martensitic matrix [30,42]. The combined effects of phase transformation strain and thermal contraction, therefore, provide strong driving forces for martensite refinement and substructure fragmentation.

Following low-temperature tempering after DCT, the martensitic lath boundaries become progressively blurred and less distinct, and a characteristic corrugated or wrinkled substructure develops within the martensite laths, as shown in Figure 7(c5–c8). The average martensite lath width is further reduced to approximately 97 nm, indicating that the refinement effect is not only preserved but further enhanced after tempering. This morphological evolution can be attributed to recovery processes during tempering, including partial dislocation annihilation, rearrangement, and polygonization, as well as the stabilization and redistribution of fine cementite precipitates. The interaction between high dislocation density and fine cementite particles effectively suppresses lath coarsening and promotes the formation of a stable, refined tempered martensitic substructure.

The formation of such refined and stabilized tempered martensitic structures is well known to effectively reduce internal stresses while maintaining a high density of strengthening defects, thereby preserving high strength and hardness while improving toughness and structural stability. Consequently, the combined DCT and subsequent tempering produce a hierarchical microstructure characterized by refined martensite laths, high dislocation density, and uniformly distributed cementite, which is highly beneficial for achieving a favorable balance between hardness, wear resistance, and toughness in microalloyed high-carbon steels [43,44,45].

### 3.2. Mechanical Properties After Different Heat Treatments

**Figure 8:**
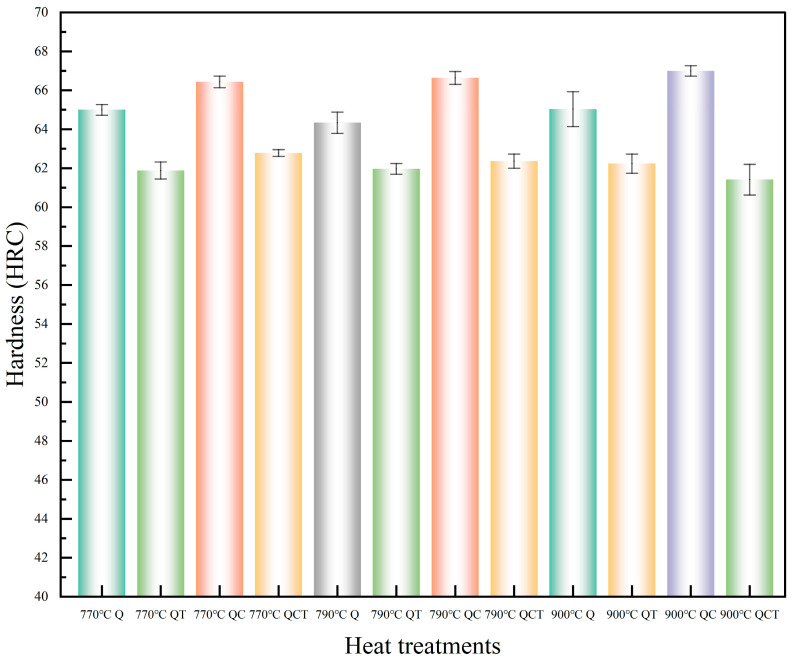
Hardness variation after different heat treatments.

**Figure 9:**
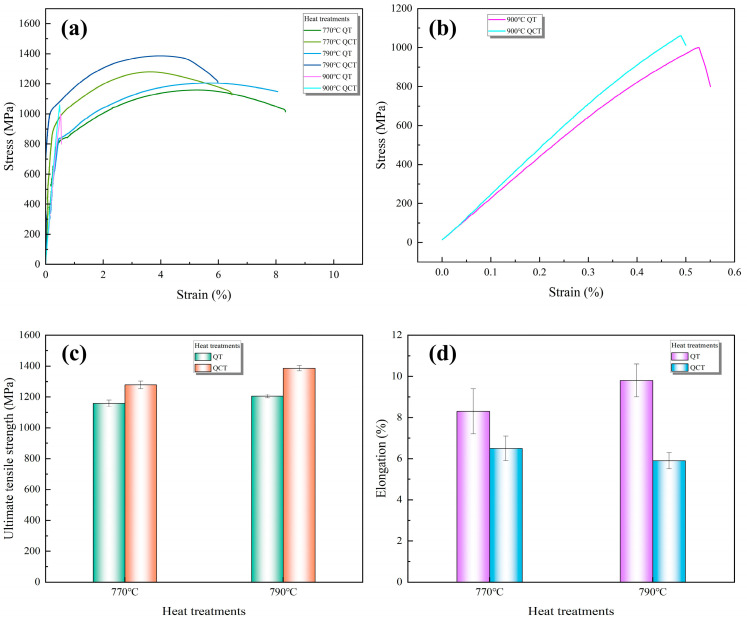
Tensile properties of the microalloyed high-carbon steel under different heat treatment conditions: (**a**,**b**) stress–strain curve; (**c**) ultimate tensile strength; (**d**) elongation.

Figure 8 and Figure 9 present the variations in hardness and tensile properties of the microalloyed high-carbon steel subjected to different heat treatment routes. As shown in Figure 8, the hardness of the quenched specimens increases as the austenitizing temperature rises from 770 °C to 900 °C, which can be attributed to the enhanced dissolution of alloy carbides and the corresponding increase in carbon content in austenite before quenching. The subsequent DCT further increases the hardness of the as-quenched specimens, primarily due to the transformation of retained austenite into martensite and the associated increase in martensitic volume fraction. After tempering, a reduction in hardness is observed for all conditions, reflecting the relief of internal stresses and partial recovery of the martensitic matrix.

From the perspective of tensile properties (Figure 9a–d), the ultimate tensile strength (UTS) of the specimens increases after quenching at 770–790 °C, and DCT leads to a further enhancement in UTS. This strength increment is closely related to the combined effects of higher martensite carbon supersaturation, reduced retained austenite content, and a more uniform dispersion of fine secondary carbides induced by DCT. However, both increasing the austenitizing temperature and applying DCT result in a noticeable decrease in elongation. This trade-off behavior arises from the increased amount of dissolved carbides and the higher level of carbon in solid solution at elevated austenitizing temperatures, which promote martensite strengthening while simultaneously reducing the capacity for uniform plastic deformation.

When the austenitizing temperature was increased to 900 °C, the steel exhibited an almost complete loss of macroscopic ductility and failed in a brittle manner during tensile testing, without significant yielding or necking. This pronounced embrittlement can be attributed to the synergistic effects of multiple factors, including severe austenite grain coarsening, increased carbon supersaturation in the martensite, and localized stress concentration at weakened grain boundaries, rather than being solely caused by grain growth alone. Austenitizing at 900 °C results in a microstructure characterized by coarse prior austenite grains and highly supersaturated martensite, both of which are detrimental to ductility. The extensive carbide dissolution at this temperature promotes rapid grain coarsening, thereby reducing the grain boundary area available for effective crack deflection. Moreover, the increased carbon in solid solution elevates the tetragonality and internal stress of the as-quenched martensite. Consequently, the steel exhibits severely enhanced brittleness and a compromised capacity for plastic deformation.

During subsequent DCT, stresses arising from retained austenite transformation and cryogenic volumetric contraction localize severely at grain boundaries, which have been weakened by prior grain coarsening and the dissolution of pinning phases. The interplay between these localized tensile stresses and the intrinsically brittle high-carbon martensite promotes preferential intergranular crack initiation and rapid propagation. The brittle fracture observed after austenitizing at 900 °C is therefore the outcome of a cascade of detrimental effects. Coarsened grains undermine boundary strengthening and resistance to crack growth, while carbon supersaturation elevates lattice strain and intrinsic brittleness. These conditions are compounded by the localization of transformation- and cryogenic-induced stresses at the already-weakened grain boundaries, culminating in catastrophic intergranular fracture [46].

### 3.3. Fracture Morphology Analysis

**Figure 10:**
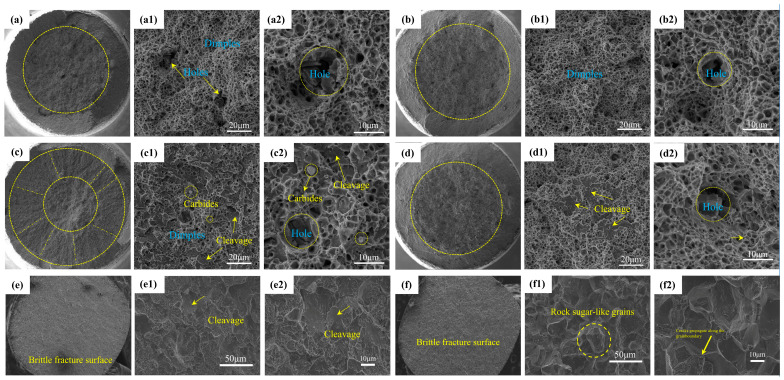
Fracture morphology of tensile specimens after different heat treatments: (**a**) Macroscopic fracture morphology of 770 °C QT; (**a1**,**a2**) Microscopic morphology of 770 °C QT; (**b**) Macroscopic fracture morphology of 770 °C QCT; (**b1**,**b2**) Microscopic morphology of 770 °C QCT; (**c**) Macroscopic fracture morphology of 790 °C QT; (**c1**,**c2**) Microscopic morphology of 790 °C QT; (**d**) Macroscopic fracture morphology of 790 °C QCT; (**d1**,**d2**) Microscopic morphology of 790 °C QCT; (**e**) Macroscopic fracture morphology of 900 °C QT; (**e1**,**e2**) Microscopic morphology of 900 °C QT; (**f**) Macroscopic fracture morphology of 900 °C QCT; (**f1**,**f2**) Microscopic fracture morphology of 900 °C QCT.

Figure 10 presents the tensile fracture morphologies of the experimental steel subjected to different heat treatments. Figure 10a,c,e show the fracture surfaces of QT specimens, and the corresponding high-magnification images are provided immediately thereafter; for example, Figure 10(a1,a2) display the detailed fracture morphology of the specimen quenched at 770 °C and subsequently tempered. Figure 10b,d,f illustrate the fracture surfaces of QCT specimens, as well as Figure 10(b1,b2) showing the high-magnification features of the QCT specimen treated at 770 °C. The same sequence applies to the other austenitizing conditions.

As shown in Figure 10a,c, the specimens quenched at 770 °C and 790 °C and subsequently tempered exhibit pronounced fibrous zones, radial marks, and well-developed shear lips, all of which are characteristic features of macroscopic ductile fracture. However, the relatively limited reduction in area reflects the intrinsic plastic constraint associated with high-carbon steels. At higher magnification, the fracture surfaces of the quenched and tempered specimens are dominated by dimples, indicating that microvoid nucleation, growth, and coalescence constitute the primary fracture mechanism.

In comparison with the QT specimens, the DCT-treated samples (Figure 10b,d) exhibit finer and more uniformly distributed dimples, consistent with the slightly improved elongation observed in the tensile tests. This behavior suggests that DCT enhances local plastic deformation capacity through microstructural refinement and a more homogeneous dispersion of carbides [47]. In addition, cracked or fractured carbide particles are observed on the fracture surfaces, indicating that carbides act as brittle phases and preferential sites for microcrack initiation during tensile deformation. Notably, finer secondary carbides are detected in the DCT-treated specimens, which can be attributed to carbon redistribution and nanoscale carbide precipitation induced during cryogenic treatment [26,48]. For the specimen austenitized at 790 °C and subjected to DCT, small cleavage facets are locally observed, indicating an increased tendency toward brittle fracture. This observation is consistent with the simultaneous decrease in tensile strength and elongation under this condition.

In sharp contrast to the mixed fracture mode observed at lower austenitizing temperatures, both the QT and QCT specimens austenitized at 900 °C exhibit a typical intergranular “rock-candy” fracture morphology (as shown in Figure 10e,f), characterized by flat facets and distinct grain boundary separation, indicative of a fully brittle fracture mode.

This pronounced embrittlement is primarily attributed to the detrimental effects of excessive austenitizing temperature.

First, severe austenite grain coarsening significantly reduces the effective grain boundary area and the resistance to crack propagation. Second, prolonged exposure to high temperature promotes the dissolution of grain boundary pinning phases and intensifies impurity segregation, thereby weakening grain boundary cohesion. Under such conditions, the highly localized transformation stresses and associated volume expansion introduced during subsequent DCT, together with the intrinsic brittleness of high-carbon martensite, preferentially concentrate along these weakened grain boundaries. Previous studies on high-carbon steels have confirmed that the combined effect of microstructural degradation and processing-induced stresses ultimately leads to catastrophic intergranular cracking [49,50].

These results highlight a critical limitation of the DCT process: although DCT is beneficial under optimized austenitizing conditions, its effectiveness is significantly reduced or even reversed when applied to a microstructure already embrittled by excessive grain growth. The present study demonstrates that combining DCT with an appropriate austenitizing temperature (e.g., 770 °C) effectively refines martensite, reduces retained austenite, and promotes the precipitation of finely dispersed carbides, thereby achieving a favorable balance between strength and ductility. In contrast, excessive austenitizing (e.g., 900 °C) leads to microstructural coarsening and grain boundary embrittlement, resulting in catastrophic brittle fracture. Accordingly, the dominant fracture mechanism transitions from carbide-controlled microvoid coalescence under optimized conditions to intergranular brittle fracture at excessively high austenitizing temperatures.

## 4. Discussion

### 4.1. Influence of Microalloying on the Precipitated Phases

**Figure 11:**
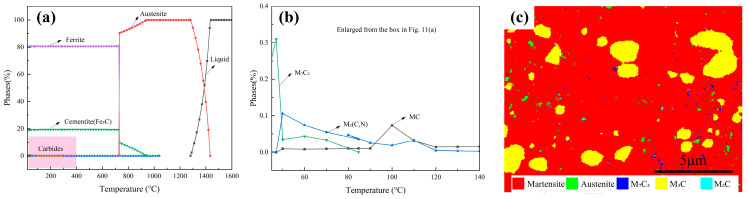
Calculated thermodynamic equilibrium phases in the investigated steel and corresponding EBSD validation: (**a**,**b**) mole fraction of equilibrium phases as a function of temperature; (**c**) EBSD phase map identifying the types and distribution of precipitates.

The equilibrium precipitation behavior of the experimental steel was assessed through thermodynamic calculations, and the predicted stable phases are shown in Figure 11a,b. The results indicate that the addition of microalloying elements, particularly Mo, V, and Ti, markedly modifies the phase stability of the high-carbon steel [51]. Under equilibrium conditions, several alloy carbides, including M_7_C_3_, MC, and M_2_C, are thermodynamically favored. These carbides are typical of microalloyed steels containing strong carbide-forming elements and are widely recognized for their ability to restrict austenite grain growth, enhance precipitation strengthening, and improve microstructural stability [52]. However, it should be noted that equilibrium calculations represent an idealized condition requiring sufficiently long holding times and extensive diffusion, which differs substantially from the actual heat-treatment schedules employed in this study.

As is well known, the specific processing routes, including quenching, DCT, and low-temperature tempering, all have a strong kinetic constraint effect on the precipitation of carbides in practice [53]. Consequently, TEM observations reveal that cementite (Fe_3_C) is the dominant carbide, while well-developed equilibrium alloy carbides are not readily detected. Nevertheless, EBSD phase analysis (Figure 11c) provides complementary evidence for the presence of M_7_C_3_, M_2_C, and M_3_C carbides, suggesting that alloy carbides can form locally in an extremely fine or metastable state. This discrepancy reflects the different sensitivities of TEM and EBSD and indicates that microalloying elements participate primarily through kinetic regulation rather than equilibrium precipitation. By partially partitioning into cementite or remaining in supersaturated solid solution within martensite, these elements influence carbide stability and precipitation kinetics, particularly during DCT and subsequent tempering. As a result, a refined martensitic substructure with finely dispersed carbides is obtained, which contributes directly to the improved strength and dimensional stability of the microalloyed high-carbon steel [54].

### 4.2. Effect of Austenitizing Temperature on Microstructural Evolution

**Figure 12:**
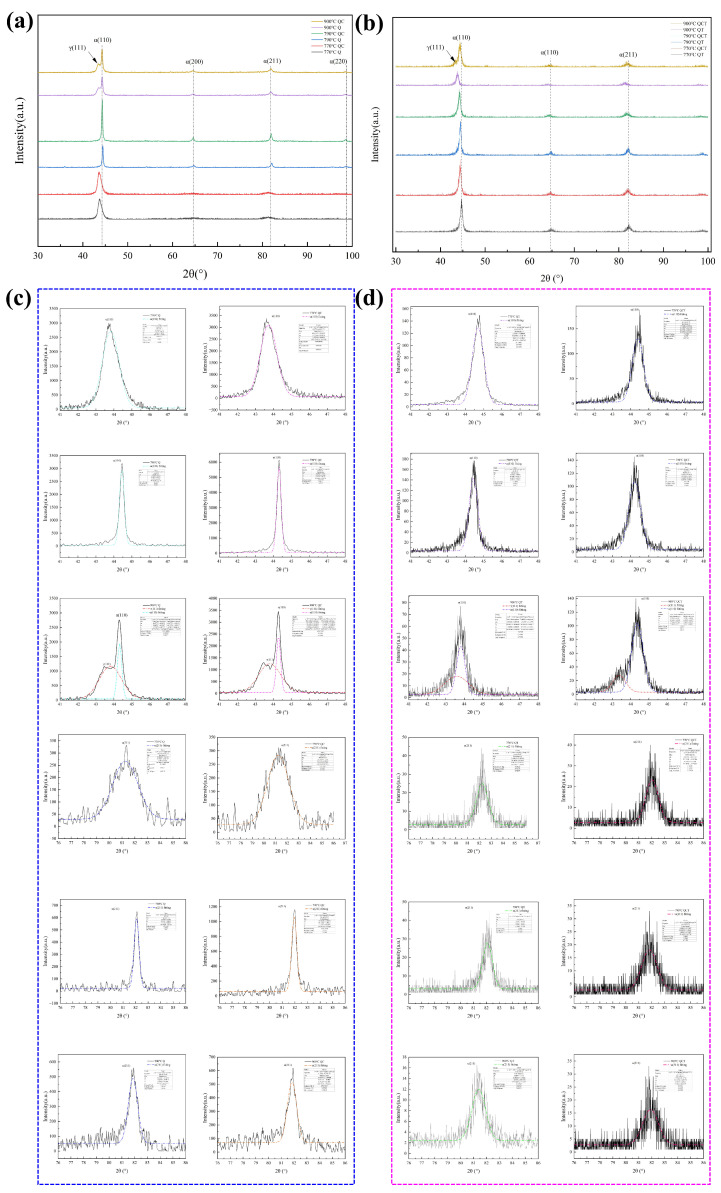
X-ray diffraction analysis after different heat treatments: (**a**) diffraction patterns of the Q and QC samples; (**b**) fitting results of the α(110) and α(211) diffraction peaks for Q and QC; (**c**) diffraction patterns of the QT and QCT samples; (**d**) fitting results of the α(110) and α(211) diffraction peaks for QT and QCT.

Figure 12 presents the XRD patterns of the experimental steel subjected to different heat-treatment routes at various austenitizing temperatures, together with the fitted profiles of the martensitic α(110) and α(211) diffraction peaks. Specifically, Figure 12a,c show the XRD patterns of the Q and QC specimens corresponding to different austenitizing temperatures, along with the fitted α(110) and α(211) peaks. Figure 12b,d display the corresponding results for the QT and QCT specimens. In addition, the XRD patterns of the specimens after quenching and deep cryogenic treatment are included, with detailed peak fitting of the martensitic reflections to evaluate phase constitution and lattice characteristics.

It can be seen that at relatively low austenitizing temperatures (770 °C and 790 °C), the diffraction patterns are dominated by α-Fe (martensite) peaks, while no obvious γ-Fe (austenite) diffraction peaks are detected. This indicates that the majority of austenite transformed into martensite during quenching. It should be noted, however, that when the retained austenite content is below approximately 5 vol.%, the phase resolution of conventional XRD becomes limited, and small amounts of retained austenite may remain undetected.

When the austenitizing temperature increases to 900 °C, a weak γ(111) diffraction peak appears as a shoulder on the α(110) peak, revealing the presence of a small amount of retained austenite. The emergence of the γ phase at higher austenitizing temperatures can be attributed to the enhanced dissolution of carbides and the increased carbon enrichment in austenite, which improves its thermodynamic stability and suppresses complete martensitic transformation during cooling [55]. Beyond phase identification, the intensity and position of martensitic diffraction peaks exhibit pronounced dependence on the austenitizing temperature.

As illustrated in Figure 12a,c, the intensity of the α(211) diffraction peak increases noticeably with increasing austenitizing temperature, while the α(110) peak becomes sharper and steeper, indicating an evolution in crystallographic texture and lattice distortion. When the austenitizing temperature increases from 770 °C to 790 °C, both the α(110) and α(211) peaks shift toward higher diffraction angles (2θ). According to Bragg’s law, such a shift corresponds to a reduction in the interplanar spacing and thus a decrease in the effective lattice parameter. In high-carbon martensitic steels, this behavior can be associated with enhanced dissolution of carbides at elevated austenitizing temperatures, leading to increased carbon enrichment in austenite before quenching. The subsequent martensitic transformation results in a higher degree of carbon supersaturation and intensified lattice distortion, which modifies the diffraction condition and contributes to the observed peak shift. After DCT, the martensitic peak intensities increase, reflecting the further transformation of retained austenite into martensite and an increase in the overall martensitic volume fraction. Meanwhile, the overall 2θ positions exhibit a slight tendency to shift toward lower angles, suggesting subtle changes in lattice strain and carbon redistribution during cryogenic exposure. This behavior is consistent with stress redistribution and the formation of a more homogeneous martensitic structure.

However, compared with the specimens austenitized at 790 °C, those treated at 900 °C show a marked decrease in martensitic peak intensity, despite the near-complete dissolution of cementite at this higher temperature. This apparent anomaly can be attributed to the combined effects of increased retained austenite fraction, partial stress relaxation, and possible changes in crystallographic texture. Together, these factors reduce the effective diffracting volume of martensite and weaken the corresponding diffraction peak intensity. This result further indicates that excessively high austenitizing temperatures alter not only phase fractions but also the internal strain state and orientation distribution of martensite, thereby significantly affecting the XRD response.

The XRD patterns of the tempered specimens, together with the fitted α(110) and α(211) peaks, are presented in Figure 12b,d. After tempering, the microstructure of all specimens remains predominantly martensitic, regardless of the prior austenitizing temperature, indicating that low-temperature tempering does not significantly alter the phase constitution but mainly modifies the internal lattice state. With increasing austenitizing temperature, the martensitic peaks of the QT specimens exhibit a gradual shift toward lower diffraction angles. This behavior reflects partial carbon redistribution from supersaturated martensite to carbides during tempering, accompanied by the relaxation of transformation-induced lattice strain. In contrast, the martensitic peaks of the QCT specimens are generally located at slightly higher diffraction angles than those of the corresponding QT specimens. This difference suggests that prior deep cryogenic treatment promotes a more effective redistribution of carbon and stabilizes the lattice distortion inherited from quenching, thereby retaining a relatively higher level of lattice strain after tempering and suppressing excessive recovery.

Overall, the XRD results indicate that the austenitizing temperature plays a dominant role in determining the carbon state, lattice distortion, and stability of retained austenite, whereas DCT acts as an important regulator of martensitic lattice strain and carbon redistribution during subsequent tempering.

### 4.3. Role of DCT and Tempering on Microstructure Evolution

**Figure 13:**
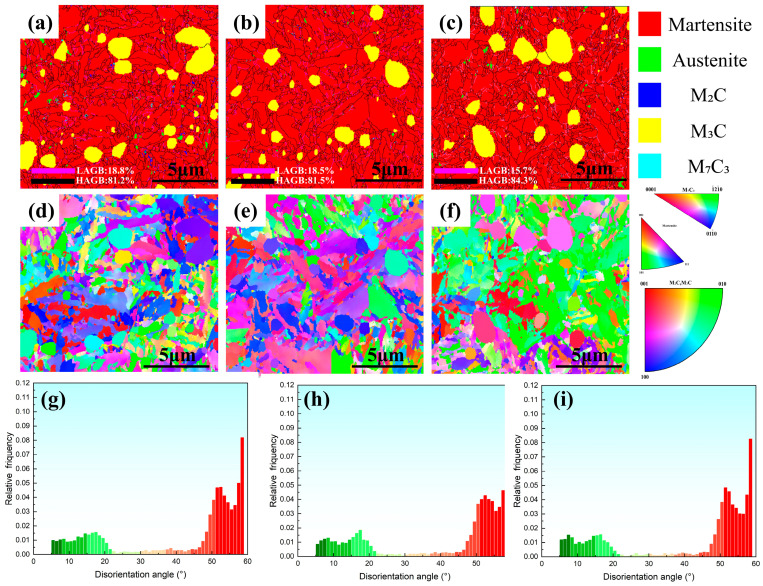
EBSD analysis of grain boundary character and crystallographic orientation evolution under different heat treatment conditions: (**a**–**c**) Phase maps overlaid with grain boundaries. Low-angle grain boundaries (LAGBs, 2° ≤ θ < 15°) are depicted in pink, while high-angle grain boundaries (HAGBs, θ ≥ 15°) are shown in dark. (**d**–**f**) Inverse pole figure (IPF) maps along the normal direction (ND) of the sample, illustrating the crystallographic orientation distribution within the microstructure. (**g**–**i**) Corresponding misorientation angle distribution histograms.

Figure 13 presents the EBSD analysis of phase constitution, grain boundary characteristics, and crystallographic orientation evolution of the specimens austenitized at 790 °C under different heat-treatment conditions (Q, QC, and QCT). Specifically, Figure 13a,d,g correspond to the Q specimen, Figure 13b,e,h to the QC specimen, and Figure 13c,f,i to the QCT specimen. As shown in Figure 13a–c, the microstructure after quenching at 790 °C is predominantly composed of martensite and cementite (Fe_3_C), with only a minor fraction of retained austenite. Quantitative EBSD phase analysis indicates that the volume fraction of retained austenite remains below 1%. This finding is consistent with the absence, or extremely weak intensity, of austenite diffraction peaks in the XRD patterns, which may be attributed to the limited detection sensitivity of XRD for phases present in very low volume fractions. In addition to Fe_3_C, trace amounts of M_7_C_3_ and M_2_C-type carbides are also identified, suggesting that microalloying elements participate in localized carbide formation even under non-equilibrium heat-treatment conditions. Following DCT and subsequent tempering, a small amount of retained austenite is still detectable within the microstructure. This retained austenite is primarily distributed along high-angle grain boundaries, where local chemical enrichment, strain partitioning, and stress accommodation collectively enhance its mechanical and thermal stability. The persistence of retained austenite after DCT and tempering indicates that not all austenite transforms during cryogenic cooling; regions stabilized by carbon enrichment and constrained by the surrounding martensitic matrix remain untransformed. Such stabilized retained austenite may contribute to stress relaxation and microstructural stability during service [56,57].

The evolution of grain boundary characteristics is further illustrated by the grain boundary maps in Figure 13a–c. The fraction of low-angle grain boundaries (LAGBs, 2–15°) decreases marginally from 18.8% in the quenched condition to 18.5% after DCT, and further to 15.7% after DCT followed by tempering. The slight reduction in LAGB fraction after DCT can be attributed to the transformation of retained austenite, which introduces fresh dislocations and promotes the reorganization of existing sub-boundaries. The more pronounced decrease observed after tempering reflects recovery processes, including dislocation rearrangement and annihilation, which lead to the gradual elimination of low-angle boundaries and the establishment of a more stabilized boundary network.

Figure 13d–f presents the inverse pole figure (IPF) maps of the experimental steel under the three processing conditions. The IPF maps reveal a distinct grain structure with irregular polygonal morphologies and a wide grain size distribution, indicative of an intrinsically heterogeneous martensitic microstructure. Both coarse and fine martensite packets coexist, reflecting the combined influence of prior austenite grain size variation and transformation kinetics. A comparison of the IPF maps for the quenched and quenched + DCT conditions (Figure 13d,e) shows that DCT does not substantially alter the overall crystallographic orientation relationship; the martensite orientations remain randomly and uniformly distributed. This suggests that DCT primarily influences defect configurations and phase stability rather than inducing texture evolution.

Notably, after DCT followed by tempering, an increased fraction of martensite grains with {101} orientation is observed. This orientation enrichment can be attributed to selective recovery and stabilization during tempering, wherein martensite variants possessing lower stored energy and more favorable slip systems are preferentially retained. The redistribution of internal stresses and carbon during tempering promotes the stabilization of specific crystallographic orientations, leading to the observed increase in {101}-oriented martensite.

The misorientation angle distributions shown in Figure 13g–i further illustrate the evolution of boundary characteristics. The relative frequency of boundaries plotted as a function of misorientation angle reveals a gradual shift toward lower average misorientation following DCT. This reduction in misorientation is associated with the fragmentation of martensite substructures and the reorganization of dislocation configurations during DCT, accompanied by enhanced sub-boundary formation and a more homogeneous distribution of lattice curvature. Such microstructural evolution contributes to improved strength through dislocation hardening while simultaneously enhancing microstructural stability.

**Figure 14:**
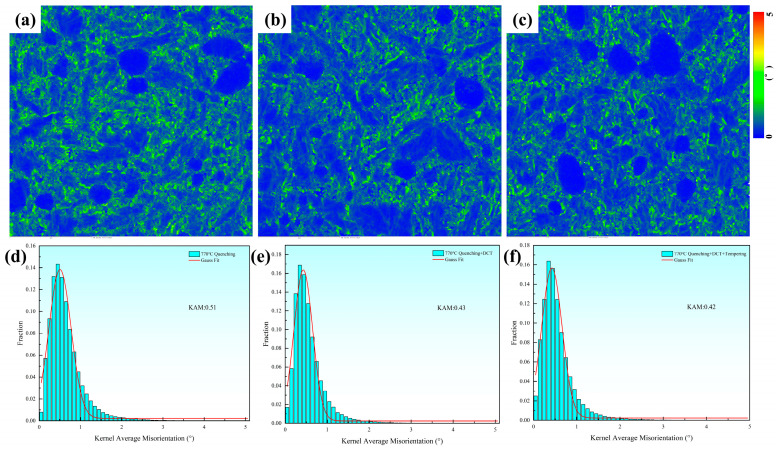
KAM maps and corresponding KAM value distributions of the experimental steel under different heat-treatment conditions (austenitized at 790 °C): (**a**,**d**) Q; (**b**,**e**) QC; (**c**,**f**) QCT.

Figure 14 presents the kernel average misorientation (KAM) maps and the corresponding statistical distributions of KAM values for the experimental steel subjected to different heat-treatment routes. In the KAM maps, the color contrast represents the degree of local lattice distortion and stress concentration, which is commonly associated with the density of geometrically necessary dislocations (GNDs). As clearly observed in Figure 14a–c, regions with relatively high KAM values (green-colored areas) are mainly concentrated near phase boundaries, particularly at the interfaces between carbides and the martensitic matrix, as well as within fine martensitic grains. This phenomenon can be attributed to the strong strain incompatibility arising from the elastic modulus mismatch and transformation-induced volume expansion between hard carbides and the surrounding martensite. Moreover, smaller grains possess a higher boundary area and accommodate deformation through increased dislocation accumulation, resulting in enhanced local lattice curvature and elevated KAM values.

For the specimen quenched at 790 °C, the average KAM value reaches 0.51°, indicating a high density of dislocations introduced during the rapid martensitic transformation and subsequent thermal contraction. After DCT, the average KAM value decreases significantly to 0.43°, suggesting an effective relaxation and redistribution of internal stresses. This reduction can be attributed to the combined effects of retained austenite transformation, which homogenizes strain distribution, and cryogenically enhanced carbon redistribution that promotes the formation of fine, dispersed carbides capable of pinning dislocations and mitigating severe dislocation pile-up at phase interfaces. After subsequent tempering, the average KAM value further decreases slightly to 0.42°. This additional reduction reflects the recovery and reorganization of dislocation structures during tempering, accompanied by partial annihilation and stabilization of the martensitic substructure. Tempering also facilitates the release of residual stresses accumulated during quenching and DCT, leading to a more uniform lattice strain state.

## 5. Conclusions

(1) Moderate austenitizing (770–790 °C) can yield fine laths of martensite and limited retained austenite, while excessively high temperatures (900 °C) lead to coarsening of austenite grains, widening of martensite laths, and an increase in retained austenite fraction, thereby reducing the uniformity and stability of the microstructure.

(2) DCT can promote the transformation of retained austenite to martensite, improve the carbon distribution, further refine the martensite laths, and at the same time increase the dislocation density and redistribute the local strain. Subsequent low-temperature tempering further reduces the width of martensite lathes, forming a uniform tempered martensite substructure, thereby improving toughness while maintaining strength.

(3) The main role of microalloying elements (V, Ti, Mo) is reflected in the inhibition of grains and the slight contribution of carbide precipitation during austenitizing and quenching processes. Under the conditions of this study, the amount of MC-type carbides formed is extremely small, and their direct strengthening effect on mechanical properties is limited. The austenitizing temperature and the regulation of martensite transformation and carbide precipitation by cryogenic treatment mainly determine the material’s macroscopic strength and toughness.

## Figures and Tables

**Table 1:** Chemical composition of the steel (wt.%).

Element	C	Si	Mn	Cr	Ni	Mo	V	Ti	S	P	Fe
wt.%	1.15–1.24	≤0.35	≤0.40	≤0.25	≤0.04	0.01	0.002	0.003	≤0.03	≤0.01	Bal.

## Data Availability

The original contributions presented in this study are included in the article. Further inquiries can be directed to the corresponding author.

## References

[1] Jurči P., Orihel P., Keddam M., Vretenár V., Maťko M. (2025). Boride layers on high-carbon high-chromium tool steels: Microstructure-mechanical properties relationship. J. Mater. Res. Technol..

[2] Kosiba K., Wolf D., Bönisch M., Neufeld K., Hühne R., Gustmann T., Bednarčík J., Chen H., Han X., Hoffmann V. (2023). Achieving exceptional wear resistance in a crack-free high-carbon tool steel fabricated by laser powder bed fusion without pre-heating. J. Mater. Sci. Technol..

[3] Heydari A., Zarei-Hanzaki A., Mahmoudi M., Moshiri A., Jaskari M., Karjalainen L.P., Balanian H., Abedi H.R. (2024). Small crack growth behavior and correlated microstructure-sensitivity during cyclic deformation of a high-chromium/carbon wrought tool steel. Mater. Sci. Eng. A.

[4] Wang B., Zhu D., Zhang C., Zhou X., Wu H., Wang S., Wu G., Gao J., Zhao H., Mao X. (2025). Influence of typical elements and heat treatment parameters on hardenability in steel: A review. J. Iron Steel Res. Int..

[5] Yin L., Zhang T., Xv F., Yu Y., Zhang S., Wang Y. (2026). Microstructure-dependent tribocorrosion mechanisms of low-carbon martensitic stainless steel. Wear.

[6] Pinson M., Springer H., Depover T., Verbeken K. (2021). The effect of quench cracks and retained austenite on the hydrogen trapping capacity of high carbon martensitic steels. Int. J. Hydrogen Energy.

[7] Hui W., Borysenko A.Y., Klemeshov Y.S., Yan G., Ambrazhey M.Y., Malysh O.D. (2025). High-temperature strength and plasticity of medium- and high-carbon steels. Mater. Sci..

[8] Liu T., Zhang X., Cui X., Chen S., Sun X., Long J., Zheng Z. (2025). Effects of C content and tempering temperature on impact-abrasive wear resistance of high-C martensitic steel. J. Iron Steel Res. Int..

[9] Agnani M., Findley K.O., Thompson S.W. (2024). Effects of retained austenite and martensite microstructure on fatigue crack propagation in quenched and tempered high carbon steels. Int. J. Fatigue.

[10] Hossain R., Pahlevani F., Sahajwalla V. (2017). Effect of small addition of Cr on stability of retained austenite in high carbon steel. Mater. Charact..

[11] Hossain R., Pahlevani F., Sahajwalla V. (2019). Stability of retained austenite in high carbon steel—Effect of post-tempering heat treatment. Mater. Charact..

[12] Bała P., Gajewska M., Cios G., Kawałko J., Wątroba M., Bednarczyk W., Dziurka R. (2022). Effect of GA+ ion beam on the stability of retained austenite in high carbon steel. Mater. Charact..

[13] Song Y., Zhang C., Cheng Z., Chen Y., Wang S., Zhu D., Wu H. (2025). Fatigue properties of microalloyed steels: A review. J. Iron Steel Res. Int..

[14] Zhao L., Zhai G., Wu J., Chen X., Zhai Q. (2025). Microstructure and mechanical properties of a novel Nb–V–Ce multi-microalloyed low-alloy cast steel. J. Iron Steel Res. Int..

[15] Lu C., Liu X., Cao J., Zhang Y., Wang Z., Zhou X., Xu C., Gan Z., Zhao W. (2025). Achieving a high synergy of strength, ductility, and toughness through Nb microalloying in high-carbon pearlite steels. Mater. Today Commun..

[16] Zhang C., Hu Q., Zhang S., Peng Z., Li M., Liu J. (2025). Synergistic regulation of mechanical properties and pitting corrosion resistance of high-nitrogen austenitic stainless steel via vanadium microalloying. J. Iron Steel Res. Int..

[17] Yu Y., Liu H., Zhang S., Zhang C., Liu X., Sun Z., Zhang J., Chang C. (2025). Achieving a trade-off of high strength and low thermal expansion in invar steel via synergy of dislocations and vanadium carbonitrides. J. Iron Steel Res. Int..

[18] Wang Y., Che Z., Chen Y., Yang S., Zhang J., Xue Q. (2025). Strength and toughness mechanism of single Ti microalloyed steels. J. Iron Steel Res. Int..

[19] Zhang M., Zhang T., Hu C., Geng S., Jia L., Hu F., Zhang J., Zhao J., Misra R.D.K., Wu K. (2025). Synergistic effects of V, Ti, and Nb microalloying on prior austenite grain growth kinetics in high-carbon pearlitic steel wire rods. J. Mater Res. Technol..

[20] Wang D., Xiao J., Cao K., Yang S., Zhao A. (2025). Evolution of NbC from nucleation and precipitation to growth and coarsening in Nb-microalloyed high-carbon martensitic steel. Mater. Lett..

[21] Dey I., Saha R., Ghosh S.K. (2019). Influence of Microalloying in High Carbon Pearlitic Steel. Mater. Today Proc..

[22] Li J., Zhang X., Bu H., Qi H., Zuo P., Li S., Li M. (2023). Effects of deep cryogenic treatment on the microstructure evolution, mechanical and thermal fatigue properties of H13 hot work die steel. J. Mater. Res. Technol..

[23] Kara F., Küçük Y., Özbek O., Özbek N.A., Gök M.S., Altaş E., Uygur İ. (2023). Effect of cryogenic treatment on wear behavior of Sleipner cold work tool steel. Tribol Int..

[24] Jovičević-Klug P., Puš G., Jovičević-Klug M., Žužek B., Podgornik B. (2022). Influence of heat treatment parameters on effectiveness of deep cryogenic treatment on properties of high-speed steels. Mater. Sci. Eng. A.

[25] Li D., He W., Zhang X., Xiao M., Li S., Zhao K., Yang M. (2021). Effects of traditional heat treatment and a novel deep cryogenic treatment on microstructure and mechanical properties of low-carbon high-alloy martensitic bearing steel. J. Iron Steel Res. Int..

[26] Li S., Xiao M., Ye G., Zhao K., Yang M. (2018). Effects of deep cryogenic treatment on microstructural evolution and alloy phases precipitation of a new low carbon martensitic stainless bearing steel during aging. Mater. Sci. Eng. A.

[27] Li J., Tang L., Li S., Wu X. (2013). Finite element simulation of deep cryogenic treatment incorporating transformation kinetics. Mater. Des..

[28] Li J., Feng Y., Tang L., Wu X. (2013). FEM prediction of retained austenite evolution in cold work die steel during deep cryogenic treatment. Mater. Lett..

[29] Li S., Min N., Li J., Wu X., Li C., Tang L. (2013). Experimental verification of segregation of carbon and precipitation of carbides due to deep cryogenic treatment for tool steel by internal friction method. Mater. Sci. Eng. A.

[30] Gavriljuk V.G., Theisen W., Sirosh V.V., Polshin E.V., Kortmann A., Mogilny G.S., Petrov Y.N., Tarusin Y.V. (2013). Low-temperature martensitic transformation in tool steels in relation to their deep cryogenic treatment. Acta Mater..

[31] Jovičević-Klug P., Tegg L., Jovičević-Klug M., Parmar R., Amati M., Gregoratti L., Almásy L., Cairney J.M., Podgornik B. (2023). Understanding carbide evolution and surface chemistry during deep cryogenic treatment in high-alloyed ferrous alloy. Appl. Surf. Sci..

[32] Jovičević-Klug P., Jovičević-Klug M., Podgornik B. (2020). Effectiveness of deep cryogenic treatment on carbide precipitation. J. Mater. Res. Technol..

[33] He X., Lü X., Wu Z., Li S., Yong Q., Liang J., Su J., Zhou L., Li J., Zhao K. (2021). M23C6 precipitation and Si segregation promoted by deep cryogenic treatment aggravating pitting corrosion of supermartensitic stainless steel. J. Iron Steel Res. Int..

[34] Zhou C., Kou Z., Song K., Gong J., Liu P., Gao Q., Liu X., Han X., Zhang Z., Ramasamy P. (2024). Evading strength-ductility trade-off dilemma in TRIP-assisted Fe50Mn30Co10Cr10 duplex high-entropy alloys via flash annealing and deep cryogenic treatments. Acta Mater..

[35] Jovičević-Klug P., Guštin A.Z., Jovičević-Klug M., Šetina Batič B., Lebar A., Podgornik B. (2022). Coupled role of alloying and manufacturing on deep cryogenic treatment performance on high-alloyed ferrous alloys. J. Mater. Res. Technol..

[36] Gao Q., Jiang X., Sun H., Fang Y., Mo D., Li X., Shu R. (2022). Effect mechanism of cryogenic treatment on ferroalloy and nonferrous alloy and their weldments: A review. Mater. Today Commun..

[37] Thornton R., Slatter T., Ghadbeigi H. (2013). Effects of deep cryogenic treatment on the dry sliding wear performance of ferrous alloys. Wear.

[38] Essam M.A., Shash A.Y., El-Fawakhry M.K., El-Kashif E., Megahed H. (2023). Influence of micro-alloying elements and deep cryogenic treatment on microstructure and mechanical properties of S5 cold work shock resisting tool steel. Results Mater..

[39] Yan X.F., Zhang H.T., Wang R.Z., Pang G.Y. (2000). Austenite Grain Coarsening and NbC Dissolution-Precipitation Behavior in Niobium-Bearing Steel 16Mn. J. Iron Steel Res. Int..

[40] Singh G., Pandey K.N. (2022). Effect of cryogenic treatment on properties of materials: A review. J. Process Mech. Eng. Part E..

[41] Das D., Dutta A.K., Toppo V., Ray K. (2007). Effect of Deep Cryogenic Treatment on the Carbide Precipitation and Tribological Behavior of D2 Steel. Mater. Manuf. Process..

[42] Tyshchenko A.I., Theisen W., Oppenkowski A., Siebert S., Razumov O.N., Skoblik A.P., Sirosh V.A., Petrov Y.N., Gavriljuk V.G. (2010). Low-temperature martensitic transformation and deep cryogenic treatment of a tool steel. Mater. Sci. Eng. A.

[43] Gavriljuk V.G., Sirosh V.A., Petrov Y.N., Tyshchenko A.I., Theisen W., Kortmann A. (2014). Carbide Precipitation During Tempering of a Tool Steel Subjected to Deep Cryogenic Treatment. Metall. Mater. Trans. A.

[44] Pellizzari M., Menegante V., Villa M., Somers M.A.J. On the Influence of Deep Cryogenic Treatment on Tempering Transformations in AISI D2 Steels. Proceedings of the 26th IFHTSE Congress of Heat Treatment and Surface Engineering.

[45] Xie C., Zhou L., Min N., Wu X. (2017). Effect of deep cryogenic treatment on carbon segregation in Cr8Mo2SiV tool steel during tempering. Philos. Mag. Lett..

[46] Srinivas M., Malakondaiah G., Armstrong R.W., Rama Rao P. (1991). Ductile fracture toughness of polycrystalline armco iron of varying grain size. Acta Metall. Mater..

[47] Amini K., Araghi A., Akhbarizadeh A. (2015). Effect of Deep Cryogenic Heat Treatment on the Wear Behavior of Carburized DIN 1.7131 Grade Steel. Acta Metall. Sin. Engl..

[48] Ramesh S., Bhuvaneshwari B., Palani G.S., Mohan Lal D., Mondal K., Gupta R.K. (2019). Enhancing the corrosion resistance performance of structural steel via a novel deep cryogenic treatment process. Vacuum.

[49] Zheng H., Zuo X., Wan J., Rong Y., Chen N. (2024). Intrinsic mechanism of grain size effect and grain boundary misorientation angle effect on crack propagation in martensitic steels. Eng. Failure Anal..

[50] Šolić S., Podgornik B., Leskovšek V. (2018). The occurrence of quenching cracks in high-carbon tool steel depending on the austenitizing temperature. Eng. Failure Anal..

[51] Samanta B., Siriwardane E.M.D., Akr D. (2024). Ab initio prediction of phase stability of quaternary Mo1xMxAlB (M=Cr, Fe, Mn, Nb, Sc, Ta, Ti, V, and W) MAB solid solutions. J. Appl. Phys..

[52] Rey T. (2017). Investigation of Microstructure and Mechanical Properties in Hot-work Tool Steels. Master’s Thesis.

[53] Yuan X.B., Wu Y.W., Zhong M., Ma J.J., Kaldre I., Wang C. (2025). Influence of cooling rate upon weld metal microstructural evolution behaviors of EH36 shipbuilding steel. J. Iron Steel Res. Int..

[54] Zhang Q., Wang J., Zhou M., Liu M., Gan X., Xu G. (2025). Review on the Microalloying of Niobium, Vanadium, and Titanium in High-Carbon Steels. Steel Res. Int..

[55] Chang J., Wang M., Yang Y., Wu Y., Mi Z. (2025). Modeling of austenite formation kinetics overlapping recrystallization in cold-rolled Q&P steel during ultrafast heating process. J. Iron Steel Res. Int..

[56] Montaña Y., Idoyaga Z., Iza-Mendia A. (2025). Thermomechanical processing during warm deformation in a medium C microalloyed steel for developing spheroidised and ultrafine grained microstructures. J. Iron Steel Res. Int..

[57] Timokhina I.B., Hodgson P.D., Pereloma E.V. (2004). Effect of microstructure on the stability of retained austenite in transformation-induced-plasticity steels. Metall. Mater. Trans. A.

